# The Quality of Mobile Apps Used for the Identification of Pressure Ulcers in Adults: Systematic Survey and Review of Apps in App Stores

**DOI:** 10.2196/14266

**Published:** 2020-06-16

**Authors:** Janine Koepp, Miriam Viviane Baron, Paulo Ricardo Hernandes Martins, Cristine Brandenburg, Ariane Tieko Frare Kira, Vanessa Devens Trindade, Luis Manuel Ley Dominguez, Marcelo Carneiro, Rejane Frozza, Lia Gonçalves Possuelo, Marcus Vinicius De Mello Pinto, Liane Mahlmann Kipper, Bartira Ercília Pinheiro da Costa

**Affiliations:** 1 University of Santa Cruz do Sul Santa Cruz do Sul Brazil; 2 Pontifical Catholic University of Rio Grande do Sul Porto Alegre Brazil; 3 Coordination of Improvement of Higher Education Personnel – Brazil Brasília Brazil; 4 School of Medicine Pontifical Catholic University of Rio Grande do Sul Porto Alegre Brazil; 5 Federal University of Ceará Fortaleza Brazil; 6 National Council for Scientific and Technological Development – Brazil Brasilia Brazil; 7 Celulare Institute Rio de Janeiro Brazil

**Keywords:** software, portable app, mobile app, pressure sore, decubitus ulcer, wounds and injuries

## Abstract

**Background:**

The increasing global use of smartphones has contributed to the growing use of apps for various health conditions, showing promising results. Through mobile apps, it is possible to perform chronological and iconographic follow-up of wounds, such as pressure ulcers, using a simple and practical tool. However, numerous surveys have pointed out issues related to the functionality, design, safety, and veracity of app information.

**Objective:**

The objective of this study was to perform a systematic review of published studies regarding mobile apps and a systematic survey in app stores looking for apps developed to identify, evaluate, treat, and/or prevent pressure ulcers in adults, and to evaluate those apps based on software quality characteristics.

**Methods:**

This review followed Preferred Reporting Items for Systematic Reviews and Meta-Analyses (PRISMA) guidelines. The main bibliographic databases were searched between January 1, 2007 and October 15, 2018, and an app survey was performed in app stores. The selected studies were evaluated according to software quality characteristics by the International Organization for Standardization/International Electrotechnical Commission (ie, ISO/IEC 25010:2011) that involve functionality, efficiency, compatibility, usability, reliability, safety, maintenance, and portability.

**Results:**

The search in databases and web-based app stores returned a total of 2075 studies. After removal of duplicates and screening of titles and abstracts, 48 complete articles were evaluated for eligibility, and among these, six were included for qualitative synthesis.

**Conclusions:**

In this review, it was observed that all studies involved the initial phase of app development or improvement, and therefore, the apps still need to be evaluated using different software quality characteristics, so that in the future, a gold standard can be approached. Therefore, the prescription of an app for the identification, evaluation, treatment, and/or prevention of pressure ulcers in adults is currently limited. However, the evaluated studies provided important insights for future research. It is of utmost importance that future surveys develop apps jointly with users, using collaborative and cocreative processes and assess patients in real-world situations across different service settings, and they should consider different ethnicities, so that apps are useful to end users, such as patients, family members, health professionals, and students, in the health area. In addition, it is necessary for studies to describe the methodological course of app development in a clear and objective way in order to ensure reproducibility of the study and to offer inputs to allow future research to approach the development of ideal apps that are geared to positively impact the health of end users.

**Trial Registration:**

PROSPERO CRD42018114137; https://www.crd.york.ac.uk/prospero/display_record.php?RecordID=114137

## Introduction

### Background

A pressure ulcer involves localized damage to the underlying skin or soft tissues, resulting in localized tissue destruction related to lack of blood flow due to increased external pressure on bone prominence or due to the use of a medical device [1].

Pressure ulcers negatively impact the quality of life of patients, contribute to pain and suffering, prolong hospitalization, increase workload, and increase costs for health systems [2-9]. In addition, the incidence and prevalence rates remain high in different populations and countries. Studies in the intensive care units (ICUs) of hospitals in Brazil showed an incidence of pressure ulcers between 17.2% and 41.0% [10,11]. In the United States, the prevalence of pressure ulcers in the ICU ranges from 8.8% to 12.1%, and in acute care units, it can reach 22% [12].

A pressure ulcer is a wound that is characterized by rapid deterioration of soft tissues and a process of chronification that hinders normal healing. In this way, systematic follow-up of the evolution of the wound by a physician and health team is unavoidable. However, evaluation and follow-up by specialized professionals in loco are not always possible, especially in situations where the patient cannot count on special transportation to a specialized care center or the patient does not have a family member or resident in remote areas. With the emergence of mobile health (mHealth) and the popularity of mobile devices in clinics and hospitals, wound evaluation can now be optimized by allowing an interprofessional team to remotely view, analyze, and monitor wound evolution through apps [13].

A recent study has shown that through an app developed for the management of pressure ulcers, it is possible for a caregiver to show the patient a digital image of a wound on the buttocks, the back region, or under the foot. In this way, the approach brings to patients and families a better understanding of the wound and subsequent compliance with wound treatment guidelines. In addition, app image algorithms can calculate the size of the wound, and additionally, color analysis can aid in the detection of the depth and stage of the pressure ulcer [2], facilitating the monitoring of the evolution of the wound and the choice of correct treatment. Another app developed for pressure ulcer prevention allows users to monitor the pressure of a seat interface in real time, leading to pressure relief maneuvers and allowing the transfer of monitored data for follow-up by a specialist [9]. Advances in this area have shown that apps can assist health professionals in the prevention and treatment of pressure ulcers and facilitate the involvement of family members and patients in their own care, improving management and wound outcomes [2].

Currently, there is an overall increase in app use for various health conditions [14-30]. The convenience in using apps is associated with the many resources available through smartphones. With these facilities, the user may acquire skills and confidence and may adapt quickly to the use of the tool [23]. However, researchers have analyzed apps geared toward the management of various health problems and have discovered several problems with regard to navigability, usability, functionality, design, accuracy, unnecessary resources, lack of free apps, and lack of certification of the quality of the information conveyed, and most apps access personal data on devices. These shortcomings have cast doubts on the applicability and efficacy of mobile apps in various health care sectors [16,18,26,27,29,31,32].

Therefore, knowing if mobile apps used to track pressure ulcers have quality features like functionality, reliability, usability, efficiency, compatibility, security, maintenance, and portability is of extreme relevance to end users. In this context, the research question was elaborated in the format of the acronym PICO (participants, intervention, comparison, outcomes) and was formulated as follows: do mobile apps used by adults to identify, evaluate, treat, and/or prevent pressure ulcers present software quality characteristics?

### Objective

To answer the research question, the aim of this study was to conduct a systematic review of published studies on mobile apps and a systematic survey in app stores for apps developed to identify, evaluate, treat, and/or prevent pressure ulcers in adults, as well as to evaluate apps based on software quality characteristics.

## Methods

### Review Protocol

We used the Preferred Reporting Items for Systematic Reviews and Meta-Analyses (PRISMA) guidelines for the design of this study, as well as to report the findings of the review [33]. The protocol of this systematic review was registered in the International Prospective Register of Systematic Reviews (PROSPERO; ID: CRD42018114137).

### Search Strategy in Databases and App Stores

A literature search was carried out in collaboration with librarians with experience in systematic reviews. For the search and selection of studies, the following databases were selected: PROSPERO, PubMed, Cochrane Library, Cumulative Index to Nursing and Allied Health Literature (CINAHL), Web of Science, Institute of Electric and Electronic Engineering (IEEE) Xplore Digital library, Compendex (Ei Village 2), Association for Computing Machinery (ACM) Digital Library, Science Direct, Scopus, Scientific Electronic Library Online (SciELO), Literatura Latino-americana e do Caribe em Ciências da Saúde (Latin American and Caribbean Health Sciences Literature database) (LILACS), Google Scholar, and Brazilian Registry of Clinical Trials (ReBEC). The search strategy with key words was initially developed for PubMed (Textbox 1) and later adapted to the other databases according to the syntax required in each database. The online search was carried out from October 6 to November 5, 2018. Additional bibliographies were searched in the references of relevant studies, with contacting of authors and search for gray literature. Additionally, a search was performed for the names of apps about pressure ulcers in web-based stores and subsequently for queries that involved the names of the apps found.

**PubMed search strategy.**

((“nursing”[Subheading] OR “nursing”[All Fields] OR “nursing”[MeSH Terms] OR (“patient care team”[MeSH Terms] OR (“patient”[All Fields] AND “care”[All Fields] AND “team”[All Fields]) OR “patient care team”[All Fields])) AND (“sensitivity and specificity”[MeSH Terms] OR (“sensitivity”[All Fields] AND “specificity”[All Fields]) OR “sensitivity and specificity”[All Fields])) OR accuracy[All Fields]) AND (“android”[All Fields])) OR (iOS[All Fields]) OR (“mobile applications”[MeSH Terms] OR (“mobile”[All Fields] AND “applications”[All Fields]) OR “mobile applications”[All Fields])) OR (“mobile”[All Fields] AND “app”[All Fields]) OR “mobile app”[All Fields])) OR (“cell phone”[MeSH Terms] OR (“cell”[All Fields] AND “phone”[All Fields]) OR “cell phone”[All Fields])) OR (“smartphone”[MeSH Terms] OR “smartphone”[All Fields])) AND (“pressure ulcer”[MeSH Terms] OR (“pressure”[All Fields] AND “ulcer”[All Fields]) OR “pressure ulcer”[All Fields])) OR (“decubitus”[All Fields] AND “ulcers”[All Fields]) OR “decubitus ulcers”[All Fields])

### Eligibility Criteria

We considered studies published on the internet in English, Portuguese, or Spanish, dated between January 1, 2007 and October 15, 2018. The search was updated on November 5, 2018. It included original research work limited to humans; studies involving apps on handheld devices having Android, iOS, or other operating systems; studies that resulted in the development of software registration; studies with mobile apps that aimed to identify, evaluate, treat, and/or prevent pressure ulcers; studies involving app users, such as health professionals and information technology professionals, over the age of 18 years; studies that used apps to identify, evaluate, treat, and/or prevent pressure ulcers in individuals over the age of 18 years; and studies performed in care settings, such as university, hospital, and community settings.

The exclusion criteria were as follows: review studies; studies involving mobile apps that, in addition to identifying, evaluating, treating, and/or preventing pressure ulcers, assessed other types of wounds or other health problems; studies involving other electronic information systems; studies involving only electronic forms and electronic medical records; and studies where key information was not available. In the case of duplicate studies, the search considered those with a larger sample size and more information.

### Selection Process and Data Extraction

All records were downloaded to Mendeley Desktop Version 1.19.3 and duplicates were removed. All the titles and summaries of the remaining studies were read to identify whether they met the eligibility criteria. Whenever titles and summaries were not sufficient, the full text of the potentially relevant studies was read in its entirety. Two researchers performed the entire process of searching the databases, selecting the studies, and reading the studies independently.

**Figure 1:**
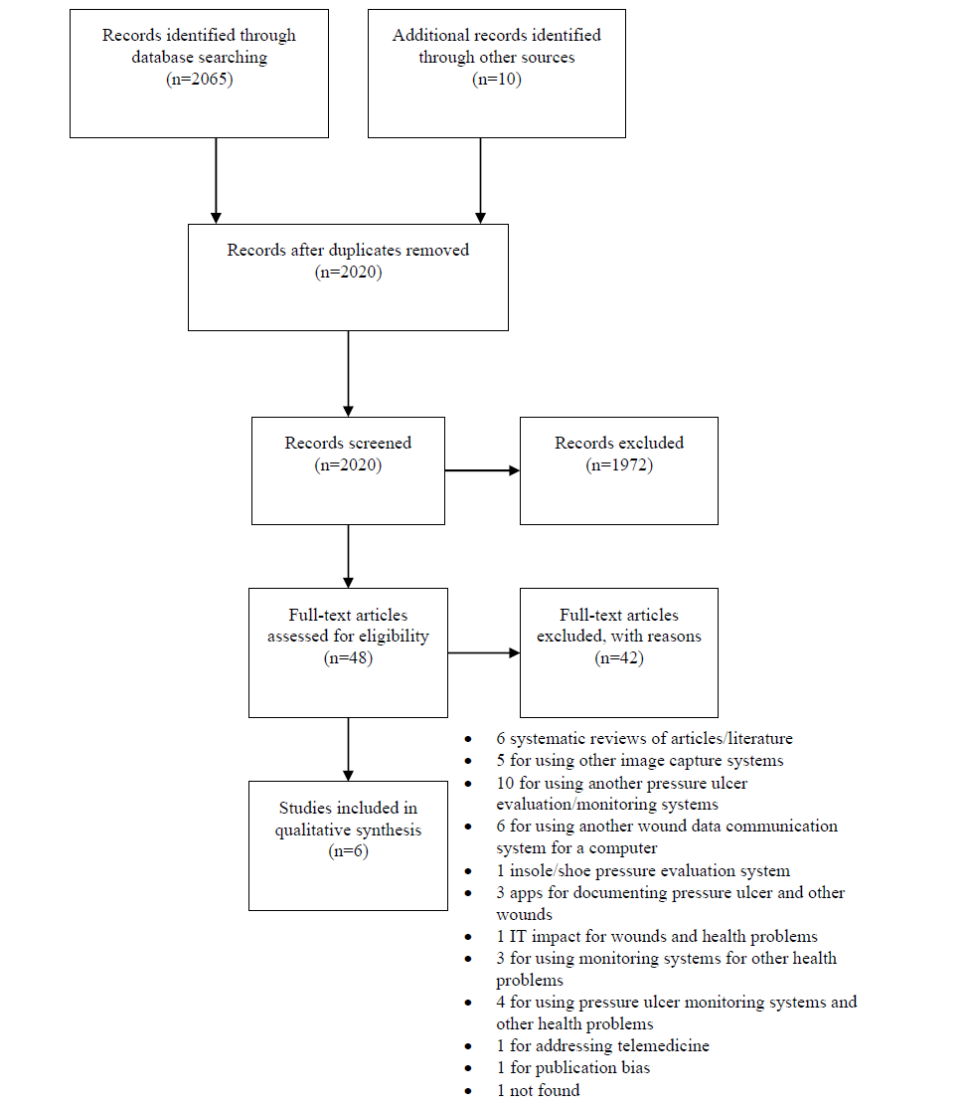
PRISMA flowchart.

Thereafter, the two authors independently extracted data from the selected studies using a standardized Word table template. The following data were extracted from the studies: author and year of publication, study outline, objective, sample, description of the technology, main results, and characteristics of the quality of the app. Disagreements were resolved by consultation and discussion with a third senior author to reach consensus. The screening and selection of studies are presented in a PRISMA flow diagram (Figure 1).

### Data Synthesis and Quality Assessment

A narrative and qualitative synthesis was performed. As a summary of the main result metrics, we evaluated any type of result that indicated or measured app quality. This evaluation was based on the standards of the International Organization for Standardization (ISO)/International Electrotechnical Commission (IEC) 25010:2011 called Software Product Quality Requirements and Evaluation (SQuaRE). ISO/IEC 25010:2011 is prescribed for the evaluation of software in production or already developed. This evaluation is based on quality characteristics, such as functionality, efficiency, compatibility, usability, reliability, safety, maintenance, and portability [34].

The original protocol aimed at evaluating any type of study outcome that indicated or measured the accuracy of mobile apps used for the identification, evaluation, treatment, and/or prevention of pressure ulcers, and evaluating the methodological quality of the studies using the Downs and Black instrument (1998) [35]. However, all the included studies involved descriptive searches with apps in the initial development or improvement process (ie, in the preclinical stage of innovation), which did not allow us to evaluate the accuracy of the apps. Therefore, there was a deviation from the original protocol.

## Results

### Search Results

A total of 2065 studies were initially identified in the database search, and nine studies were additionally identified through a search of the references of the studies that met the inclusion criteria, through a search of the gray literature, and contact with authors. In addition, the search for apps on pressure ulcers on websites and web-based stores identified 18 apps (Table 1).

During the search for names, in order to find published research on the development of apps, two studies were screened; however, only one was retrieved, resulting in a total of 2075 assessed studies. After the removal of duplicate studies and screening of the titles and abstracts, 48 full-text articles were evaluated for eligibility. Of those 48 studies, six were included for qualitative synthesis. The qualitative synthesis of these six studies selected for this review can be found in Table 2. It should be noted that the distribution of the studies by year of publication shows that these six studies were published within the last 10 years.

**Table 1:** List of apps found on websites and in web-based stores.

App	Description	Capture photos of the wound (yes/no)	Research involving the app (yes/no)	Developer	Platform	Device	Language	Paid or free	Country of origin
Wound Rounds	PU^a^ prevention, showing data over the internet	No	No	Tele Medicine Solutions, LLC	iOS	Tablet and smartphone	English	Paid	USA
Staging PI	PU classification	No	No	Baylor Scott & White Health	iOS and Android	Tablet and smartphone	English	Free	USA
Pressure Ulcer	PU prevention	No	No	Patient Data Science, LLC	iOS and Android	Tablet and smartphone	English	Free	USA
Pressure Ulcer Guide	PU prevention	No	No	Patient Data Science, LLC	iOS and Android	Tablet and smartphone	English	Paid	USA
Braden Scale 4 Pressure Ulcer	PU prevention using Braden scale	No	No	Patient Data Science, LLC	iOS and Android	Tablet and smartphone	English	Paid	USA
Norton Scale 4 Pressure Ulcer	PU prevention using Norton scale	No	No	Patient Data Science, LLC	iOS and Android	Tablet and smartphone	English	Paid	USA
MOWA	Identifying, assessing, and suggesting PU care	Yes	Yes	Healthpath	iOS and Android	Tablet and smartphone	English, Italian, Spanish, French, and Portuguese	Paid	Italy
PrevenAPP	PU prevention using Braden scale	No	No	Smith & Nephew	INA^b^	INA	No longer available	Free	UK
Riesgo de Úlceras Por Presión (Pressure Ulcer Risk)	PU prevention using Braden scale	No	No	Luis Miguel Delgado	Android	Tablet and smartphone	Spanish	Free	Colombia
GuíaUPP	Provides information and tools for PU prevention, diagnosis, and treatment	No	Yes	ERTAKY	iOS and Android	Tablet and smartphone	Spanish	Free	Spain
SmartUPP	PU prevention and treatment	No	No	Viacore IT	iOS and Android	Tablet and smartphone	English	Free	Spain
Trata la UPP	Provides information for PU treatment	No	No	Head Life APP	Android	Tablet and smartphone	Spanish	Free	INA
VAPUR – Pressure Ulcer Resource	PU prevention	No	No	US Department of Veterans Affairs	iOS and Android	Tablet and smartphone	English	Free	USA
WoundMAP PUMP	Evaluation of PUs and development of a care plan	Yes	No	Mobile Health Ware	iOS	INA	English	INA	INA
Ulcercare	PU risk assessment and recommendations of care. Patient data can be shared over the internet	Yes	No	Dermtap	iOS	INA	English	INA	INA
Wound Mender	PU risk assessment. Patient data can be shared over the internet.	Yes	No	IOSTREAM	iOS	INA	English	INA	INA
BCX Braden	PU prevention using Braden scale	No	No	BioCapax Technologies SLU	iOS and Android	Tablet and smartphone	English	Free	Spain
Care of Sweden	PU prevention	Yes	No	Care of Sweden	iOS and Android	Tablet and smartphone	English, Swedish, Norwegian, Danish, Finnish, German, Spanish, French, and Dutch	INA	Sweden

^a^PU: pressure ulcer.

^b^INA: information not available.

**Table 2:** Studies included for the qualitative synthesis of the systematic review.

Author/year	Study design	Objective	Sample	Technology description	Main results	Characteristics of the quality of the software product ISO/IEC 25010:2011
Vos-Draper et al, 2013 [9]	Not described	Develop a prototype seat pressure mapping system through a mat that transmits data to a smartphone app in real time.	Five individuals presenting spinal cord injury and wheelchair users.	Each individual performed three separate sessions and sat on the mat for 3 consecutive hours. Pressure relief was performed for 2 min every 30 min.	Over 3 hours, the mean pressure tended to increase with time, while the dispersion index remained more constant. The app prototype did not allow the clinician to select individual scatter index areas on the map, so generic sections were used. The app allows users to self-monitor.	Functionality
Faux et al, 2016 [13]	Not described	Propose a smartphone app for wound tracking.	One individual simulates the development of a heel PU^a^ during a 7-week period of hospitalization.	A mobile app (prototype) evaluated by health care professionals in a controlled environment. Photos of wound evolution at 7 weeks were simulated using texture modeling. To preserve an unchanging angle of the camera shooting a transparent image, a mask was placed over the current capture of the photo instead of dots and lines around the wound.	The captured photos showed almost the same scale and orientation throughout the 7 weeks of the study. Quantitative results showed a variation of 40% of the area and 25% of the perimeter due to the difficulty of aligning the mask over the current image of the wound, especially in the intensive care unit or hospital room when patient mobility is reduced.	Functionality, efficiency, and usability
Tibes 2015 [36]	Applied research	Develop a mobile app prototype that assists in the prevention and classification of PUs.	Eight nursing specialists and eight computer specialists.	It used Android, Java programming language, which was provided by Android SDK^b^ and Android Studio, and the KNN^c^ algorithm. The requirement analysis was used for software development.	App navigation flowchart. The app presents the user with a list of PU care recommendations. The user can capture a photo of the PU, and the system will process this image with a suggestion of the probable stage of the injury. Additionally, it calculates the score using Braden scale.	Functionality, efficiency, usability, reliability, maintainability, and portability.
Friesen et al, 2013 [3]	Not described	Create an interface that maximizes user compliance and data value for primary users.	Eight nurses from a health unit.	The nurses received a smartphone or tablet with the app, 90-min training, and a training manual. The nurses used the app in their daily practice with at least seven consecutive shifts. After 3 weeks, web-based research was applied on the design and functionality of the app and 6 weeks after a focus group session was held.	The nurses reported that the app was logical. However, they identified the need for more cross browsing between the various areas of the app and indicated that the list of treatments section was very long. They observed that the value of the wound image depended on how the photographs were taken. Based on user testing, the researchers will work on improvements in the design and development of image analysis algorithms.	Functionality, efficiency, usability, safety, and maintainability.
Poon et al, 2015 [2]	Not described	To develop an algorithm that determines the size of the wound in relative and absolute terms and to analyze the color of the PU image.	This is the SMARTWOUNDCARE mobile app, with a description of the enhancement of image analysis algorithms.	The following three algorithms were used: mask image, camera calibration, and color analysis.	It was possible to automatically detect the size of the PU, as well as the color of the wound and to ultimately correlate the PU stage. However, it was not possible to determine the depth of the PU.	Functionality and efficiency
Pérez-Barreno et al, 2013 [31]	Not described	Develop an app with recommendations for the prevention and treatment of PUs.	This is the description of the GuíaUPP app.	A bibliographic search was carried out at the Joanna Briggs Institute, ANEDIDIC^d^, and GNEAUPP^e^, and complementary searches were carried out on articles, books, and manuals. The methodology was evaluated using the AGREE^f^ instrument.	The app addresses classification, evaluation, prevention, treatment, products, and bibliographic references. The GuiaUPP provides the best and most up-to-date evidence available on the prevention and treatment of PUs.	No quality characteristics are detailed

^a^PU: pressure ulcer.

^b^SDK: software development kit.

^c^KNN: K-nearest neighbor.

^d^ANEDIDIC: Asociación Nacional de Enfermería Dermatológica e Investigación del Deterioro de la Integridad Cutánea (National Association of Dermatology Nursing and Research of Harm to Skin Integrity).

^e^GNEAUPP: Grupo Nacional para el Estudio y Asesoramiento en úlceras por Presión y Heridas Crónicas (National Group for Study and Counseling in Pressure Ulcers and Chronic Wounds).

^f^AGREE: Appraisal of Guidelines for Research & Evaluation.

### Application Quality Assessment

The study by Tibes [36] evaluated the largest number of software quality characteristics and described functionality, reliability, usability, efficiency, maintainability, and portability. The study by Faux et al [13] evaluated functionality, usability, and efficiency. The study by Friesen et al [3] evaluated the characteristics regarding usability, efficiency, safety, maintenance, and functionality. The study by Vos-Draper et al [9] evaluated functionality, and the study by Poon et al [2] evaluated functionality and efficiency. The study by Pérez-Barreno et al [31] did not describe software quality characteristics.

## Discussion

### Principal Findings

The objective of this study was to conduct a systematic review of published studies on mobile apps and a systematic survey in web-based stores looking for apps developed to identify, evaluate, treat, and/or prevent pressure ulcers in adults, and to evaluate the apps based on software quality characteristics. The studies were evaluated based on the eight software quality characteristics recommended by ISO/IEC 25010:2011 (functionality, compatibility, reliability, usability, efficiency, maintenance, safety, and portability) [34]. Based on observation of the six selected studies, we verified the use of similar technologies but different study designs and results in the initial stages, making definite analysis of app quality impossible.

### Evaluation of Apps According to ISO/IEC 25010:2011 Quality Characteristics

Vos-Draper et al [9] developed a prototype seat pressure mapping system through a mat that transmits real-time data to a smartphone app. The pressure mat was tested for skin safety, and preliminary variables were investigated for reproducibility. In the study, the development steps of the web-based app were not described. The authors reported only that the app prototype did not allow the clinician to select dispersion index areas of the individual pressures on the map, so generic sections were used, thereby compromising app efficiency (ie, the amount of resources used in the software does not meet the user’s requirements) [34]. The authors concluded that the app was successfully developed and received by users and displayed wireless carpet pressure data on a personal smartphone, allowing users to self-monitor seat interface pressure outside the clinic setting, complying with the functionality characteristic, although the evaluation of this feature has not been described in the article. The authors suggest future tests to improve app settings and additional research to determine if the prototype can successfully modify users’ behavior in pressure relief, and there is intention to use the data provided in an individualized way. It is observed that this study is still under development. Although functionality can be considered positive by wheelchair users, it is necessary to submit the app for assessment of other quality characteristics, as the app has the potential to contribute to pressure ulcer prevention and the quality of life of wheelchair users.

According to Matthew-Maich et al [37], the successful design and development process of an app involves the continuous participation of end users. In this way, researchers and engineers will have an understanding of the context in which the solution will be used by a diverse group of end users. This also makes it possible to establish, from the onset, the specific software and hardware resources that can be considered acceptable, preferable, and compatible with the users’ needs and that will influence users’ adherence at the end of the process.

Faux et al [13] described a user-centered app design with the practical goals of usability and efficiency. The mobile app prototype was evaluated by health professionals (nurses were cited in the context of image capture) in a laboratory-controlled environment replicating in detail a hospital room or home. The experiment simulated the evolution of a pressure ulcer on the heel of a fictitious patient hospitalized in the hospital for 7 weeks. The images of wounds simulated using texture modeling were used to evaluate the app. A mask image was used in the capture of the images, and algorithms were adopted to evaluate the evolution phases of the wounds. The quantitative results indicated a variation of about 40% for the area and 25% for the perimeter of the wound image due to difficulty in aligning the mask image on the current image. The results of the laboratory experience show that evaluation characteristics, such as usability, efficiency, and functionality, are clearly insufficient in this context, and it is necessary to improve the app and perform future clinical validation with health professionals in a real-life setting with real patients. It is important for apps to be tested in real environments, because in those conditions, tests reveal information not listed during app development [38]. Researchers suggest conducting targeted surveys to assess the quality of apps in partnership with patients, health care providers, and the digital industry, according to a well-established and rigorous scientific methodology with consideration of the steps developed in the elaboration of apps, as well as their validation [38-46].

The study by Tibes [36] described the development of a mobile app prototype (UpCare) aimed to provide personalized information about each patient regarding the risk, prevention, and/or classification of pressure ulcers. The development of the app was divided into five stages, namely requirements analysis, knowledge definition, computational representation, system coding, and system evaluation. In this study, eight pressure ulcer images of only one anatomical region were initially taken from the National Pressure Ulcer Advisory Panel site for the construction of the database. The RGB (red, green, and blue) color system was used for image processing, and the K-nearest neighbor algorithm was used for classification. On providing a new pressure ulcer image to the system, the algorithm automatically identifies the most similar image in the image bank and thus can estimate the pressure ulcer stage of this image. In the end, eight nursing specialists and eight computation specialists judged the app prototype through two online questionnaires. As for the quality requirements of the app, the researcher mentioned that the following six characteristics were evaluated: functionality, reliability, usability, efficiency, maintainability, and portability. The evaluators answered the evaluation questionnaire through four case studies (two for the nursing specialists and two for the computer specialists). The case studies guided a fictitious appraisal using the app. In this way, the quality evaluations performed were compromised, as ideal evaluations should occur in real environments [38,47] and without directing results favorable to research by means of case studies. Another factor considered unfavorable is that the evaluation was performed through four case studies but the result analysis was performed jointly. For security reasons, when initializing the app, user login and password were requested; however, the security feature was not evaluated in the study. The author mentioned that the objectives for the future will be the development of the final version of the app as a product and its evaluation together with users in real practice, as well as its development for the iOS platform. Other researchers [48] note the relevance of having the app assessment performed by the final users, demonstrating that these evaluations contribute to the improvement of the app and suggestions for future work. According to Sá et al [45], when users assess an app, their interaction with the product is strengthened, often identifying needs for improvements not anticipated in the initial design.

Previous researchers [3] undertook a user test to obtain feedback on the design and functionality of the pressure ulcer monitoring app called SMARTWOUNDCARE. The app was developed for smartphones and tablets and was tested by eight nurses from a health care center. The user test encompassed a focus group and a web-based survey after a period of training and use of the app. All parameters were graded on a Likert scale with scores from 1.0 (low) to 5.0 (high). Based on the assessment, nurses reported a high degree of ease in how the app guided users to insert a new medical record (score 4.57/5.00), find an existing patient record (score 4.71/5.00), add a new wound to an existing patient record (score 4.50/5.00), evaluate a wound for the first time (score 4.57/5.00), and evaluate an existing wound that has already been evaluated (score 4.29/5.00), with strong correlations between paper forms and the app in terms of content and expected data entry with scores of 4.60 (out of 5.00) for the Braden scale and 4.57 (out of 5.00) for the Pressure Ulcer Scale for Healing. These results show that the evaluators were able to validate quality characteristics related to functionality, usability, and efficiency. To ensure the privacy of patient information, data were stored on the device instead of a central server with remote access. However, the authors did not describe whether they were successful with respect to the app security feature. Appraisers suggested improvements in the maintenance feature, thus allowing the inclusion and exclusion of app patients, but overall, reported that the app is easy to understand and navigation is in accordance with the reality experienced in a health center. In this way, the quality characteristics of the app were evaluated positively. However, the main contribution of the app was related to the incorporation of the images of wounds (photographs) in the records of the patient, with a positive impact on caregivers’ work, health professionals, the patient, and family members. On the other hand, the researchers recognized that it is necessary to develop image analysis algorithms to detect wound size and chromaticity, thus improving the reliability characteristics of the app.

This study is the continuation of previous work, where the authors proposed to improve some of the fragilities referring to the quality of pressure ulcer images in the SMARTWOUNDCARE app [2]. The research team used machine learning algorithms and an image library to correlate the color of the wound with the stage of the wound. In order to increase user robustness, all images were processed. For the improvement of the app, three different algorithms were proposed. The first component presented is a mask image; with this element, the objective was to determine the change in the image size of the pressure ulcer on referring to a prior image of the same wound. The authors reported that the errors inherent in this method are associated with the user, including the mask’s sharpness and the ability to align the mask over the wound during image acquisition. In this respect, the algorithm cannot achieve the objectives of the features of functionality and efficiency because the results obtained are not compatible with what is expected [34]. The second component is camera calibration. The grabcut algorithm plays an important role in this app as an image segmentation method. To estimate the size, the method compares the pixels from two images and the algorithm calculates the relative size change between these images. Compared to other algorithms, grabcut provides efficient results with minimal human interaction, and this constitutes the main benefit in this work. In this context, the second algorithm satisfactorily reaches the evaluation of the quality of efficiency [34]. The camera calibration method can be used in conjunction with the image mask method to obtain the actual size of the wound. However, the mask image and calibration component of the camera do not identify the depth and volume of the wound. Color analysis is the third algorithm, and it determines the range of colors present in an image. This can help determine the depth or stage of the pressure ulcer. According to the researchers, an inherent problem in this method depends to a certain extent on the user’s definition of colors, and therefore, it is recommended to use a large data set to define parameters and to associate this module with machine learning. After extracting the color, the results can be sent to a specialist system to determine the pressure ulcer stage. The authors report that future work will focus on the formation of a specialist system and the development of machine learning elements, such as the support vector machine, to help determine the pressure ulcer stage. Based on the findings mentioned by the researchers, two indicators for the evaluation of quality characteristics, such as functionality and efficiency, were identified, although none of them were described in the article.

According to Pressman [49], the quality of the product depends on the quality of the development process, so it is common for higher quality apps to go through improvements in the software development process. According to Matthew-Maich et al [37], interdisciplinary app development teams need to consider specific factors when designing, deploying, and evaluating such technologies, considering working “with” and not “for” end users. With effective and efficient evidence-based app development, mHealth solutions offer great potential for improving end-user health.

The article by Pérez-Barreno et al [31] describes the GuiaUPP app. For the development of the content of the app, the researchers carried out a bibliographic search according to the main Spanish Clinical Practice Guidelines. According to the authors, the app addresses the entire problem related to the development and prevention of pressure ulcers, which can be subdivided into classification, evaluation, treatment, products, and bibliographical references. The authors point out that the GuiaUPP app provides the best and most updated evidence available on the prevention and treatment of pressure ulcers. Presently, the app is under evaluation with the intention of being included in the Mobile Health App Catalog of the Health Quality Agency of the Council of Equality, Health and Social Policies of Spain. In the app description, there is no evidence of assessment of any of the quality features of the software. The authors [38,50] argue for the need to evaluate the quality characteristics of the software, especially those of functionality, usability and efficiency. They defend that these evaluations can contribute to improvements related to appropriate technical content, better graphic presentations, and performance of the app, pointing out to specific issues and consistency with reality. According to these authors, apps should be tested in the early versions, thus optimizing the identification of problems and improving the apps before they are marketed.

### Strengths

A positive point of this review is that it highlights the importance of a dialogue between users and researchers for the technical improvement of an app. According to Friesen et al [3], after the user test, professionals reported the importance of showing images of pressure ulcers that patients were not able to see (eg, wounds on the back of the body and under the feet), and this contributed to a better understanding by patients and family members in the fulfillment of the wound treatment guidelines. The images facilitated consultation with other health professionals and saved time by avoiding the removal and replacement of the dressing each time the doctor or specialist needed to see the wound. Based on this information, researchers [2,3] improved the algorithms of image analysis, and within this perspective, they gained new insights, so that in future phases, they may propose to incorporate artificial intelligence to assist in the classification of pressure ulcer stages. In a study carried out by Vos-Draper et al [9], important final requirements were defined for the improvement of the app prototype after discussions by a focus group.

### Limitations

There are few available apps for pressure ulcers that have research regarding their development and evaluation. In this review, only six studies were found; however, we identified a wider range of apps (commercial names) that address pressure ulcers, with only one commercial app having research regarding its development. The studies in this review show great variability in relation to the methodology used in app development. Of the six studies evaluated, only one followed a specific method of software analysis [49]. Additionally, we need to mention the absence of a gold standard for comparison of the apps. Other limitations are related to fictitious evaluations that compromise the entire process of developing the app, as it requires evaluations with real patients and without image makeup (choosing the best image).

Another weakness is the composition of the image bank of photographs of only one anatomical region, which prevents the extrapolation of the research results to other anatomical zones. Other downsides are the lack of reporting of patient data security in apps and lack of reporting of the content sources of most apps. Pérez-Barreno et al [31] highlighted the relevance of evidence-based clinical practice, and within this context, they indicated the importance of recommendations that are developed systematically and with scientific rigor for apps in order to help professionals and patients make decisions about health care more appropriately.

### Clinical Implications and Future Directions

The present review identified that studies involve the initial phase of app development or improvement; therefore, apps still need to be evaluated through software quality characteristics to improve weak or absent aspects. However, the evaluation of software quality characteristics implicitly and explicitly in studies showed important points to be considered in future research. For example, capturing photographs for the identification, evaluation, and monitoring of the process of deterioration or cure of a pressure ulcer is a primordial element in app construction; however, this is an obstacle to be overcome. It is expected that the increase in technology incorporated into smartphone cameras, such as infrared thermography, algorithm enhancement, and use of artificial intelligence, may show promising results in future research. In addition, it is suggested that future studies may assess apps with regard to the technical quality of software development, using research with a rigorous scientific methodological design and with real patients. Studies should also take into account questions regarding data security, ethical issues, and the source of content, which should be based on the best scientific evidence available. It is suggested that the app image database should be composed of photographs of different anatomical regions where pressure ulcers can develop, so that the comparison of a newly captured image will be valid. In addition, there should be access to a large database of images like big data to compare newly captured images. Moreover, it is essential that apps take into account different ethnicities, with improvement of the algorithms of image analysis for individuals with darker skin, because algorithms can misclassify pressure ulcer stages in these individuals.

### Conclusions

In this review, it was observed that all studies involved the initial phase of app development or improvement, and therefore, pressure ulcer apps still need to be evaluated using different software quality characteristics, so that in the future, a gold standard can be approached. Therefore, the prescription of an app for the identification, evaluation, treatment, and/or prevention of pressure ulcers in adults is currently limited.

However, the evaluated studies provided important insights for future research. It is of utmost importance that future surveys develop apps jointly with users through collaborative and cocreative processes and assess patients in real-world situations across different service settings, and they should consider different ethnicities so that apps are useful to end users, such as patients, family members, health professionals, and students, in the health area. In addition, it is necessary for studies to describe the methodological course of app development in a clear and objective way in order to ensure reproducibility of the study and to offer inputs to allow future research to approach the development of ideal apps that are geared to positively impact the health of end users.

## Abbreviations

ICU: intensive care unit
IEC: International Electrotechnical Commission
ISO: International Organization for Standardization
